# Analysis of the association between water iodine concentration and TyG-BMI index with thyroid diseases: a cross-sectional study in Shandong province, China

**DOI:** 10.3389/fnut.2026.1819662

**Published:** 2026-05-13

**Authors:** Chun-Hu Li, Meng Zhao, Tong Zhao, Yu-Hang Liu, Zong-Yu Yue, Zhe-Xu Zhang, Xiang-Kun Zeng, Dian-Jun Sun, Peng Liu

**Affiliations:** Center for Endemic Disease Control, Harbin Medical University, Harbin, Heilongjiang, China

**Keywords:** interaction effect, nonlinear correlation, thyroid diseases, TyG-BMI, water iodine

## Abstract

**Background:**

Water iodine (WI) is a key factor influencing human thyroid function, and the TyG-BMI index, as a new metabolic indicator, can more comprehensively reflect an individual’s metabolic status. This study aims to investigate the correlation between two potential influencing factors with thyroid diseases.

**Methods:**

To explore the associations between WI and TyG-BMI index with thyroid diseases, binary logistic regression analysis, interaction and mediation analyses were adopted to analyze linear associations. Restricted cubic spline regression was used to analyze nonlinear associations. Receiver Operating Characteristic curves and the Area Under the Curve (AUC) were utilized to evaluate the predictive accuracy of WI and TyG-BMI index for thyroid diseases.

**Results:**

In this study, among the 1,301 participants, the prevalence of thyroid diseases was significantly higher in females than in males. After adjusting for covariates, excessive WI, compared to adequate levels, was associated with higher odds of thyroiditis (OR = 1.76, 95% CI = 1.01–3.08), thyroid nodules (OR = 1.73, 95% CI = 1.16–2.58), hypothyroidism (OR = 1.94, 95% CI = 1.06–3.54), and multiple thyroid diseases (OR = 2.66, 95% CI = 1.50–4.71). Besides, TyG-BMI index in the fourth quartile (Q4) was also identified as a risk factor for thyroiditis (OR = 2.06, 95% CI = 1.13–3.76). Nonlinear analyses showed that WI and TyG-BMI had significant nonlinear correlation with thyroid nodules (*p* = 0.028; *p* = 0.042).

**Conclusion:**

This study identified significant associations between WI and TyG-BMI with the prevalence of thyroid diseases. Therefore, for the prevention and control of thyroid diseases, it should be taken that targeted comprehensive measures from environmental (WI) and individual (TyG-BMI) perspectives.

## Introduction

Thyroid diseases are still one of the most common diseases worldwide, encompassing a range of conditions that affect the thyroid, such as hypothyroidism, hyperthyroidism, thyroid nodules, and thyroid cancer (1). In recent years, the prevalence of hypothyroidism, goiter, and thyroiditis had decreased significantly, but the prevalence of hyperthyroidism and Graves’ disease is 0.74 and 0.53%, respectively, the disease burden remains significant (2). The incidence of thyroid diseases is influenced by multiple factors, including both environmental and individual factors (3). In the environment, studies had demonstrated that exposure to perchlorates can impact thyroid hormone secretion, thus affecting thyroid function (4). Iodine is essential for the synthesis of thyroid hormones, and inadequate or excessive iodine intake can lead to various thyroid diseases (2, 5). However, its distribution in the environment is highly uneven. For instance, severe and long-term iodine deficiency has been associated with an increased risk of hypothyroidism, although the implementation of universal salt iodization has markedly reduced the prevalence of iodine deficiency-related thyroid disorders in China and globally (6, 7). Furthermore, individual factors such as smoking and alcohol consumption are also risk factors for thyroid diseases (8, 9). Iodine deficiency in pregnant women can cause severe neurodevelopmental deficits and congenital goiter in their offspring (10). Excessive iodine intake can lead to thyroiditis, hyperthyroidism, hypothyroidism (11).

In addition to iodine intake, thyroid diseases are closely related to metabolic syndrome (12, 13). Obesity is associated with an increased incidence of both overt and subclinical hypothyroidism, especially in individuals who are obese at baseline (14, 15). Patients with diabetes is common thyroid dysfunction. Studies have found that approximately 48% of type 2 diabetic patients exhibit thyroid dysfunction. Furthermore, research indicates that the incidence rates of hypothyroidism and hyperthyroidism are higher in diabetic patients compared to non-diabetic patients, suggesting a close association between the two conditions (16). Insulin resistance (IR) is a crucial component of metabolic syndrome, characterized by a reduced responsiveness of target organs or tissues to insulin (17, 18). Some studies have found that the triglyceride-glucose index (TyG) is a better biomarker for IR recognition and is significantly positively correlated with hyperinsulinemia (19). Building on these findings, it is hypothesized that a potential association may exist between TyG and thyroid diseases. Supporting this notion, Zhang et al. identified a significant association between the TyG index and various thyroid disorders (20).

The TyG- Body Mass Index (TyG-BMI) index is a new indicator that combines the TyG index with BMI. The TyG-BMI index can significantly improve the assessment of IR (21). This index had been found to be highly consistent with Homeostatic Model Assessment for Insulin Resistance in assessing IR in Korean populations (22) and Chinese populations (23).

In summary, although the association between iodine and thyroid diseases has been widely recognized, existing studies have largely examined environmental exposure and individual metabolic factors in isolation. To date, no study has simultaneously evaluated the joint and interactive effects of WI and the TyG-BMI index on a comprehensive spectrum of thyroid diseases in a general population. Moreover, population-based data characterizing the nonlinear relationship between WI as a continuous variable and thyroid nodules remain lacking. Therefore, this study aims to integrate both environmental and individual dimensions to provide new insights into risk stratification for thyroid diseases.

## Methods

### Data source

A multi-stage stratified random sampling method was used in this study. Based on the available data from the most recent national IDD surveillance and water iodine surveillance in China (24), Shandong Province was selected as the survey area. In the first stage, one district/county was randomly selected from the three different water iodine regions in Shandong Province. In the second stage, one town was randomly selected in each district/county. In the third stage, according to the expected sample size, 1–2 villages were randomly selected from each town.

Questionnaire and Anthropometric Measurements: Trained investigators conducted face-to-face interviews to collect participants’ socio-demographic information, lifestyle data, medical history, and medication use. Subsequently, trained professionals measured height, weight, and waist circumference using standardized instruments.

Biological Sample Collection: Venous blood samples were collected after an overnight fast of at least 8 h. Samples were temporarily stored at −20 °C in portable freezers at the field site and subsequently transported to the central laboratory, where they were stored at −80 °C until analysis. Fasting plasma glucose (FPG) and triglyceride (TG) concentrations were measured using an automatic biochemical analyzer via enzymatic colorimetric methods. Random spot urine samples were also collected for the assessment of urinary iodine concentration.

Inclusion criteria were: (1) age 20–80 years; (2) continuous residence in the local area for ≥5 years; (3) no severe communication difficulties, voluntary participation with written informed consent. Exclusion criteria were: (1) pregnant or lactating women; (2) individuals with severe liver or kidney diseases, malignant tumors, or other major underlying conditions; (3) individuals who had been taking thyroid medications (e.g., levothyroxine, methimazole, propylthiouracil, thyroid tablets, or other thyroid hormone preparations) prior to enrollment. A total of 1,344 participants were initially recruited, and 43 were excluded based on the above criteria, resulting in a final analytic sample of 1,301 participants.

This project was approved by the Ethical Review Board of Harbin Medical University (no. hrbmuecdc20200320). Written informed consent was obtained from all participants before the survey was conducted.

### Assessment of water iodine

The water iodine samples for this study were obtained from the household drinking water of the participants. Participants were provided with plastic bottles made of iodine-free material to collect samples. When collecting tap water, it was important to avoid areas near faucet outlets, direct tap sources, or potential contaminated zones to ensure the safety and purity of the water. Groundwater samples were typically collected from the of the well. After collection, the samples were sealed and stored at a low temperature (4 °C) and the iodine content in the water was determined using the iodine-potassium iodide-arsenic sulfide titration method. Briefly, water samples were first treated with an oxidizing agent under acidic conditions to convert all iodine species to iodate. Excess potassium iodide was then added to react with the iodate, liberating free iodine. The liberated iodine was subsequently titrated with standardized sodium thiosulfate solution using soluble starch as the endpoint indicator. Water iodine concentration was calculated based on the volume of sodium thiosulfate consumed.

In this study, WI was categorized into three levels according to the national standards of China: Deficiency (<40 μg/L), Adequate (40–100 μg/L), and Excess (>100 μg/L), as defined in the Delimitation of Iodine-Deficient Areas and Iodine-Adequate Areas (WS/T 669–2020) and the Definition and Demarcation of Water-Borne Iodine-Excess Areas and Iodine-Excess Endemial Areas (GB/T 19380–2016).

### Assessment of TyG-BMI

The study blood samples were immediately stored and frozen at −20 °C before delivery and subsequently transferred to the −80 °C refrigerators for storage. The assays were completed in the laboratory of the Endemic Disease Prevention and Control Center of China CDC, Harbin Medical University. TG and FPG concentrations were measured using enzymatic colorimetric methods. The TyG index was calculated as ln [TG (mg/dL) × FPG (mg/dL) / 2]. Heights and weights were measured by trained professionals, and BMI was calculated as weight (in kilograms) divided by height (in meters) squared. The TyG-BMI was calculated as TyG × BMI (25).

### Thyroid function and thyroid nodules testing

Thyroid function indicators, including thyroid stimulating hormone (TSH), free triiodothyronine (FT3), free thyroxine (FT4), antibody to thyroidperoxidase (TPOAb) and antibody to thyroglobulin (TGAb), were determined by chemiluminescence immunoassays (Siemens Healthcare Diagnostics Inc., Tarrytown, NY, USA). The TG and FBG levels were evaluated using an automatic biochemical analyzer (HITACHI 3100) (13).

Thyroid nodules were diagnosed using B-ultrasound. The same high resolution portable (Mindray 8) thyroid ultrasound instrument (ESAOTE MyLab30 model, 7.5 MHz linear transducer; Shenzhen Mindray Biomedical Electronics Co., Ltd., Shenzhen, China) with intensive training by professional sonographers at the Fourth Affiliated Hospital of Harbin Medical University was used for all participants (26).

### Diagnostic criteria of thyroid diseases

The diagnostic criteria for thyroid diseases were as follows: (1) Hypothyroidism: TSH > 4.2 μIU/mL, FT4 < 11.5 pmol/L. (2) Hyperthyroidism: TSH < 0.27 μIU/mL, FT3 > 6.8 pmol/L or FT4 > 22.7 pmol/L. (3) Positive TPOAb: TPOAb>60 U/mL. (4) Positive TGAb: TGAb> 60 U/mL. (5) Thyroiditis: Positive TPOAb or TGAb (13).

### Covariates

A structured questionnaire was used to collect socio-demographic and lifestyle-related data from participants. These data were collected after participants had received training from on-site professional staff, which included age, gender, marital status, education level, and family annual income per capita. Marital status was categorized as single, married, and divorced. Education level was categorized as illiterate, primary school, middle school, high school, and undergraduate. Family annual income per capita was categorized as <5,000, 5,000–10,000, 10,000–30,000, 30,000–50,000, and >50,000. The variable assignment is shown in Supplementary Table S1.

Lifestyle-related data included diet, smoking, alcohol consumption, physical activity, and BMI. These five lifestyle behaviors were used to construct a composite lifestyle score (27). Diet index (pure meat diet, vegetarian diet, mixed diet), total moderate to vigorous physical activity, weight (including BMI and waist circumference), smoking, and alcohol consumption were assessed. Each variable was assigned a score of 0 or 1, where 1 represented a favorable lifestyle behavior. Participants were classified based on their total lifestyle score, where 0–2 scores were categorized as poor, 3 as moderate, and 4–5 as excellent (Supplementary Table S2).

### Statistical analysis

For normally distributed continuous variables, *t*-tests were performed. For categorical variables, chi-square tests were conducted. The study initially explored the preliminary associations of thyroid diseases using Spearman correlation analysis. A binary logistic regression model was used to estimate the odds ratios (OR) and 95% confidence intervals (CI) for the association between water iodine (categorized as <40 μg/L, 40–100 μg/L, and >100 μg/L) and TyG-BMI (categorized as quartiles) with the thyroid diseases. Three models were estimated: Model 1: No adjustments were made; Model 2: Adjustments were made for age, gender, family annual income per capita, marital status, and education level; Model 3: Adjustments were made for the variables in Model 2 plus lifestyle score.

To ensure the stability of the results, subgroup analyses and sensitivity analyses were conducted. Subgroup analyses were performed stratified by gender and age groups (≤45 years and >45 years).

Since prior studies have found an association between diabetes and thyroid diseases (14, 28), the sensitivity analysis excluded participants with diabetes to further validate the stability of the results. Furthermore, the composite lifestyle score in the original Model 3 was replaced with five individual lifestyle variables (smoking, drinking, diet, physical activity, and BMI) in multivariable logistic regression to evaluate whether the independent effects of each lifestyle factor influenced the results.

To further investigate the association between WI and TyG-BMI index with thyroid diseases, a multiplicative interaction model was constructed to explore the associations of different combinations with disease.

Restricted cubic spline (RCS) regression was used to explore potential nonlinear associations. Nonlinear associations between WI and TyG-BMI index with different types of thyroid diseases were explored while controlling for confounding factors.

Finally, the study evaluated the discriminative ability of the baseline model (adjusted for age, gender, family annual income per capita, marital status, education level, and lifestyle) and the baseline + WI + TyG-BMI index model using receiver operating characteristic (ROC) curves and calculating the area under the curve (AUC) to assess their performance. Subsequently, decision curve analysis (DCA) was used to compare clinical benefits. The plots depicted net benefit against different threshold probability values. In the decision curve, a reference line labeled “treat all” represented the maximum clinical cost, while a reference line labeled “treat none” indicated no clinical benefit. Decision curves far from these reference lines indicated greater clinical value of the predictive variables. The statistical significance level for the study was set at *p* < 0.05. All analyses were implemented using R software (version 4.3.0).

## Result

### Descriptive analysis of participators and thyroid diseases

A total of 1,301 participants were included in the study, with 599 having thyroid diseases and 702 without. The study found that thyroid patients in the region had both single and multiple disease patterns. In the single disease category, thyroid nodules were the most common, with females outnumbering males. In the multiple disease group, most were dual diseases, with thyroiditis concurrent with hypothyroidism being the most common, followed by thyroid nodules concurrent with hypothyroidism (Supplementary Table S3). Participants were grouped based on whether they had thyroid diseases. There were differences in disease distribution between genders, with females having a higher prevalence rate than males (512 vs. 85). Additionally, there were differences in educational level, lifestyle score, and WI (*p* < 0.05) (Table 1).

**Table 1:** Descriptive statistics of thyroid diseases and individual characteristics.

Variables	No thyroid disease (*n* = 702)	Thyroid disease (*n* = 599)	*t*/χ2	*P*
Age	46.29 ± 10.13	47.26 ± 9.51	−1.77	0.08
Sex			**54.49**	**<0.001**
Male	222 (31.62)	85 (14.19)		
Female	480 (68.38)	514 (85.81)		
Education			**11.52**	**0.02**
None	222 (31.62)	210 (35.06)		
Elementary school	262 (37.32)	188 (31.39)		
Middle School	59 (8.40)	37 (6.18)		
High School	26 (3.70)	18 (3.01)		
University	133 (18.95)	146 (24.37)		
Annual household income			5.20	0.27
<5,000	150 (21.37)	153 (25.54)		
5,000–10,000	154 (21.94)	136 (22.70)		
10,000–30,000	243 (34.62)	197 (32.89)		
30,000–50,000	96 (13.68)	76 (12.69)		
>50,000	150 (21.37)	153 (25.54)		
Marital status			0.86^a^	0.70
Unmarried	17 (2.42)	13 (2.17)		
Married	681 (97.01)	579 (96.66)		
Divorced	3 (0.43)	5 (0.83)		
Life scores			**20.20**	**<0.001**
Poor	5 (0.71)	6 (1.00)		
Medium	257 (36.61)	150 (25.04)		
Excellent	440 (62.68)	443 (73.96)		
Water iodine			**45.48**	**<0.001**
<40 μg/L	204 (29.06)	115 (19.20)		
40–100 μg/L	178 (25.36)	99 (16.53)		
>100 μg /L	320 (45.58)	385 (64.27)		
TyG-BMI			4.21	0.239
<184.24 (Q1)	135 (22.54)	190 (27.07)		
184.24–209.35(Q2)	151 (25.21)	174 (24.79)		
209.36–239.19 (Q3)	152 (25.38)	173 (24.64)		
>239.19 (Q4)	161 (26.88)	165 (23.50)		

Bold values indicate *P* < 0.05.

### Frequency distribution of TyG-BMI and WI

**Figure 1:**
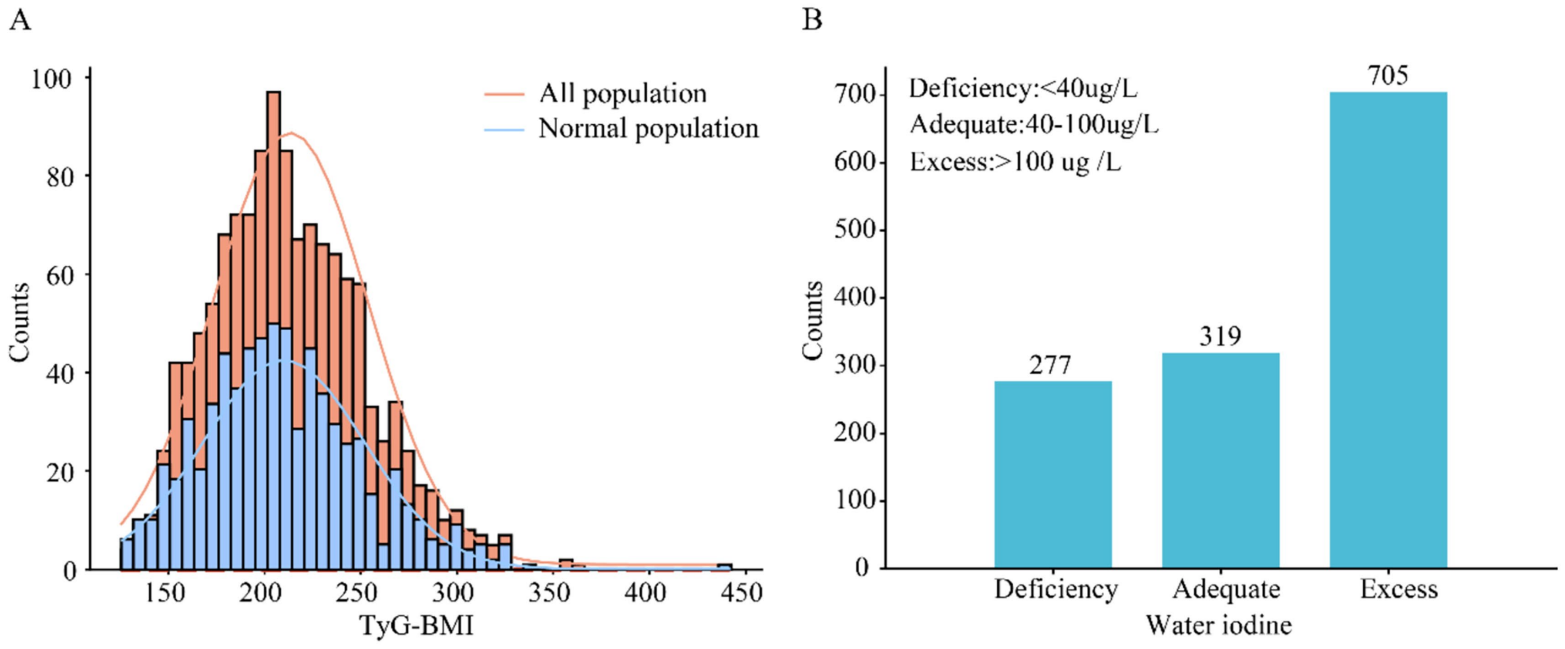
Individual TyG-BMI frequency distribution and iodine concentration in drinking water. **(A)** Frequency distribution of TyG-BMI, the orange line represents the frequency distribution of the total population, and the blue line represents the frequency distribution of the normal population; **(B)** Frequency distribution of water iodine concentration.

The TyG-BMI index showed a normal distribution trend in both the total population and the normal population (without thyroid diseases) (Kurtosis test = 3.736, *p* < 0.001; Kurtosis test = 4.049, *p* < 0.001). The 95% CI for the total population was 146.37–306.72, and for the normal population (without thyroid diseases), it was 143.17–304.23. The mean and median of TyG-BMI index values were higher in the total population compared to the normal population (Supplementary Table S4). In this study, the number of individuals with excess WI levels was the highest (*n* = 705, 54.2%), followed by Adequate (*n* = 319, 24.5%), and Deficiency (*n* = 277, 21.3%) (Figure 1).

### Spearman correlation analysis

**Figure 2:**
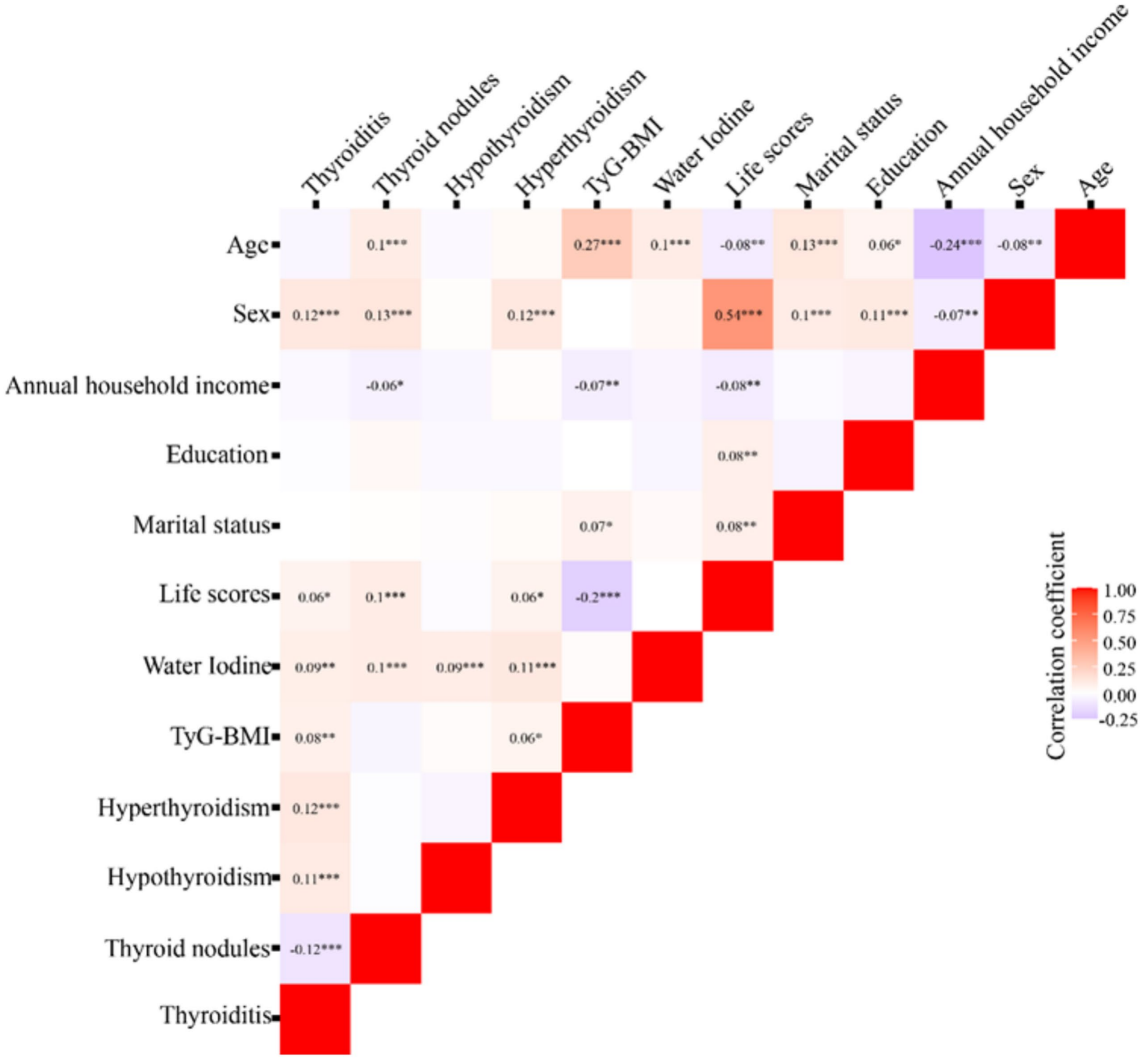
Correlation analysis of thyroid disease with various indicators. *: *p* < 0.05; **: *p* < 0.01; ***: *p* < 0.001; Red indicates a positive correlation, and blue indicates a negative correlation.

Spearman correlation analysis was conducted to explore the initial association between the variables and diseases. The results showed that thyroiditis was positively correlated with gender, lifestyle score, WI, and TyG-BMI (*r* = 0.12; *r* = 0.06; *r* = 0.09; *r* = 0.08). Thyroid nodules were positively correlated with age, gender, lifestyle, and WI, and negatively correlated with family annual income (*r* = 0.1; *r* = 0.13; *r* = 0.1; *r* = 0.1; *r* = −0.06). Hyperthyroidism was positively correlated with WI (*r* = 0.09). Hypothyroidism was positively correlated with gender, lifestyle score, WI, and TyG-BMI (*r* = 0.12; *r* = 0.06; *r* = 0.11; *r* = 0.06) (Figure 2).

Among the 1,301 participants, water iodine concentration was significantly positively correlated with urinary iodine concentration (*r* = 0.615, 95% CI: 0.571–0.656, *p* < 0.001). These findings indicate that within this study population, water iodine concentration correlates reasonably well with urinary iodine levels and may therefore reflect individual iodine nutritional status to a certain extent.

### Correlation analysis based on logistic regression model

In Model 3 of Table 2, which adjusted for multiple covariates, Excess WI was associated with increased odds of thyroiditis (OR = 1.76, 95% CI = 1.01–3.08), thyroid nodules (OR = 1.73, 95% CI = 1.16–2.58), hypothyroidism (OR = 1.94, 95% CI = 1.06–3.54), and multiple thyroid diseases (OR = 2.66, 95% CI = 1.50–4.71). TyG-BMI Quartile 4 (Q4) was a risk factor for thyroiditis (OR = 2.06, 95% CI = 1.13–3.76). After discovering that TyG-BMI Q4 was a risk factor for thyroiditis, the binary logistic regression was performed on all positive TPOAb/TGAb patients and the normal population. The results showed that TyG-BMI Q4 was a risk factor for positive TPOAb or positive TGAb compared to Q1 (OR = 1.96, 95% CI = 1.23–3.14) (Supplementary Table S5).

**Table 2:** The odds ratio and 95% confidence interval of the association between water iodine concentration and the TyG-BMI index with thyroid diseases.

Model	Variables	Classification	OR (95% CI)			
			Autothyroiditis	Thyroid nodule	Hypothyroidism	Multi-diseases
Model 1 ^a^	Water Iodine	Adequate	Ref			
		Deficiency	1.54 (0.82–2.88)	1.16 (0.74–1.81)	1.16 (0.58–2.33)	1.03 (0.53–2.02)
		Excess	**1.87 (1.06–3.28)**	**1.74 (1.18–2.57)**	**2.02 (1.11–3.66)**	**2.81 (1.62–4.88)**
	TyG-BMI	Q1	Ref			
		Q2	1.25 (0.70–2.26)	1.35 (0.91–2.01)	0.63 (0.34–1.20)	1.57 (0.89–2.78)
		Q3	1.46 (0.82–2.58)	1.13 (0.76–1.69)	0.87 (0.49–1.57)	1.41 (0.79–2.51)
		Q4	**1.89 (1.09–3.28)**	0.91 (0.60–1.38)	1.48 (0.87–2.52)	**1.80 (1.03–3.14)**
Model 2 ^b^	Water Iodine	Adequate	Ref			
		Deficiency	1.39 (0.73–2.65)	1.21 (0.76–1.92)	1.14 (0.56–2.31)	1.00 (0.50–1.99)
		Excess	1.74 (0.98–3.08)	**1.73 (1.16–2.58)**	**1.94 (1.06–3.54)**	**2.62 (1.48–4.62)**
	TyG- BMI	Q1	Ref			
		Q2	1.34 (0.73–2.47)	1.19 (0.79–1.80)	0.63 (0.33–1.21)	1.57 (0.84–2.92)
		Q3	1.61 (0.88–2.95)	0.96 (0.63–1.47)	0.88 (0.47–1.62)	1.32 (0.69–2.53)
		Q4	**2.06 (1.15–3.72)**	0.72 (0.47–1.13)	1.49 (0.84–2.64)	1.74 (0.93–3.26)
Model 3 ^c^	Water iodine	Adequate	Ref			
		Deficiency	1.39 (0.73–2.64)	1.21 (0.76–1.92)	1.14 (0.56–2.32)	0.98 (0.49–1.97)
		Excess	**1.76 (1.01–3.08)**	**1.73 (1.16–2.58)**	**1.94 (1.06–3.54)**	**2.66 (1.50–4.71)**
	TyG- BMI	Q1	Ref			
		Q2	1.34 (0.73–2.46)	1.19 (0.79–1.80)	0.62 (0.32–1.20)	1.56 (0.83–2.93)
		Q3	1.61 (0.88–2.97)	0.99 (0.64–1.52)	0.85 (0.46–1.59)	1.44 (0.75–2.79)
		Q4	**2.06 (1.13–3.76)**	0.76 (0.48–1.19)	1.41 (0.79–2.54)	1.84 (0.97–3.52)

a: No adjustment; b: Age, gender, Marital status, Annual household income, and education were adjusted on the basis of model 1; c: Life scores were adjusted based on model 2. OR: Odds Ratio; 95%CI: 95% Confidence Interval; Q1-Q4 represent the four quartiles of the TyG-BMI distribution; Multi-diseases: Individuals with two or more thyroid diseases; Bold values indicate *P* < 0.05.

### Subgroup analysis and sensitivity analysis

To further verify the associations and stability between WI and TyG-BMI with thyroid diseases, subgroup analyses and sensitivity analyses were conducted. Firstly, gender stratification was performed. In the single disease model for males, excess WI and TyG-BMI Q4 were risk factors for hypothyroidism (Supplementary Table S6). In the single disease model for females, high WI was a risk factor for thyroid nodules and concurrent multiple diseases (OR = 2.1, 95% CI = 1.32–3.35; OR = 2.81, 95% CI = 1.54–5.15). TyG-BMI Q4 remained a risk factor for thyroiditis compared to Q1 (OR = 2.30, 95% CI = 1.19–4.44). In the age stratified models, excess WI was a risk factor for thyroid nodules and hypothyroidism, and in the >45 age group, it was also a risk factor for concurrent multiple thyroid diseases (OR = 3.13, 95% CI = 1.45–6.75) (Supplementary Table S6).

Since previous studies have found an association between diabetes and thyroid disease, individuals diagnosed with diabetes in the total population were excluded, and a sensitivity analysis was conducted on the new population. In Table 3, the excess WI was a risk factor for thyroid nodules, hypothyroidism, and concurrent multiple diseases compared to adequate levels (OR = 1.74, 95% CI = 1.16–2.62; OR = 2.21, 95% CI = 1.16–4.22; OR = 2.62, 95% CI = 1.45–4.72) in the new population. The results for TyG-BMI Q4 being a risk factor for thyroiditis compared to Q1 were consistent (OR = 2.05, 95% CI = 1.12–3.74) (Table 3).

**Table 3:** Sensitivity analysis of the association between thyroid diseases with water iodine concentration and TyG-BMI index.

Variables	Classification	OR (95% CI)			
		Autothyroiditis	Thyroid nodule	Hypothyroidism	Multi-diseases
Water Iodine	Adequate	Ref			
	Deficiency	1.41 (0.74–2.68)	1.16 (0.72–1.87)	1.26 (0.60–2.67)	1.00 (0.49–2.03)
	Excess	1.64 (0.92–2.92)	**1.74 (1.16–2.62)**	**2.21 (1.16–4.22)**	**2.62 (1.45–4.72)**
TyG-BMI	Q1	Ref			
	Q2	1.31 (0.71–2.44)	1.25 (0.82–1.91)	0.77 (0.40–1.48)	1.54 (0.80–2.94)
	Q3	1.44 (0.77–2.68)	1.01 (0.65–1.57)	0.70 (0.36–1.37)	1.59 (0.82–3.09)
	Q4	**2.05 (1.12–3.74)**	0.75 (0.47–1.20)	1.53 (0.84–2.80)	1.47 (0.74–2.90)

The model population excluded patients with diabetes and was adjusted for age, gender, marital status, annual household income, education, and quality of life scores. OR: Odds Ratio; 95%CI: 95% Confidence Interval; Q1-Q4 represent the four quartiles of the TyG-BMI distribution; Multi-diseases: Individuals with two or more thyroid diseases; Bold values indicate *P* < 0.05.

Subsequently, we decomposed the components of the lifestyle score and constructed multivariable logistic regression models to evaluate whether the independent effects of individual lifestyle factors influenced the findings. As shown in Supplementary Table S7, excessive water iodine was positively associated with thyroiditis (OR = 1.80, 95% CI: 1.01–3.19), thyroid nodules (OR = 1.76, 95% CI: 1.18–2.63), hypothyroidism (OR = 1.88, 95% CI: 1.02–3.44), and multiple thyroid diseases (OR = 2.37, 95% CI: 1.70–3.30). The third and fourth quartiles of the TyG-BMI index were positively associated with thyroiditis (Q3: OR = 2.35, 95% CI: 1.16–4.75; Q4: OR = 4.02, 95% CI: 1.70–9.52), and the fourth quartile was also positively associated with multiple thyroid diseases (OR = 1.97, 95% CI: 1.14–3.39).

### Interaction analysis and mediating effects of TyG-BMI and WI

To further investigate their joint effects, a multiplicative interaction and mediation analysis were conducted. Table 4 and Supplementary Figure S1 showed the interaction results: compared to excess WI and TyG-BMI Q4, a combination of TyG-BMI Q1 and Deficiency WI was a protective factor for thyroiditis (*β* = −1.34, *p* < 0.05). Additionally, TyG-BMI Q1 + Excess WI and TyG-BMI Q2 + Adequate WI (*β* = −0.85, *p* < 0.05; *β* = −1.50, *p* < 0.05) were also protective factors. Thus, this also further demonstrates that excess WI and a higher TyG-BMI index Q4 were risk factors for thyroid disease.

**Table 4:** The interaction between TyG-BMI index and water iodine concentration on thyroid diseases.

Variables		Estimate (β)			
TyG-BMI	Water iodine	Autothyroiditis	Thyroid nodule	Hypothyroidism	Multi-diseases
Q4	Excess	Ref			
	Deficiency	−0.32	0.09	−0.62	−0.78
	Adequate	−0.90	0.51	−0.73	−0.35
Q1	Deficiency	−1.34*	0.35	−0.77	−1.87**
	Adequate	−0.64	−0.69	−1.06	−1.70*
	Excess	−0.85*	0.69*	−0.43	−0.32
Q2	Deficiency	−0.56	0.10	−1.21*	−0.70
	Adequate	−1.50*	0.09	−1.15*	−1.14*
	Excess	−0.47	0.95**	−1.04*	−0.09
Q3	Deficiency	−0.38	0.33	−1.37*	−1.40*
	Adequate	−0.79	−0.13	−1.59*	−1.40*
	Excess	−0.39	0.59*	−0.41	0.02

The model adjusted for the following variables: age, gender, marital status, annual household income, education, and life scores. OR: Odds Ratio; 95%CI: 95% Confidence Interval; Q1–Q4 represent the four quartiles of the TyG-BMI distribution; Multi-diseases: Individuals with two or more thyroid diseases. *P < 0.05, **P < 0.01, ***P < 0.001.

### Non-linear association of TyG-BMI with thyroid diseases

**Figure 3:**
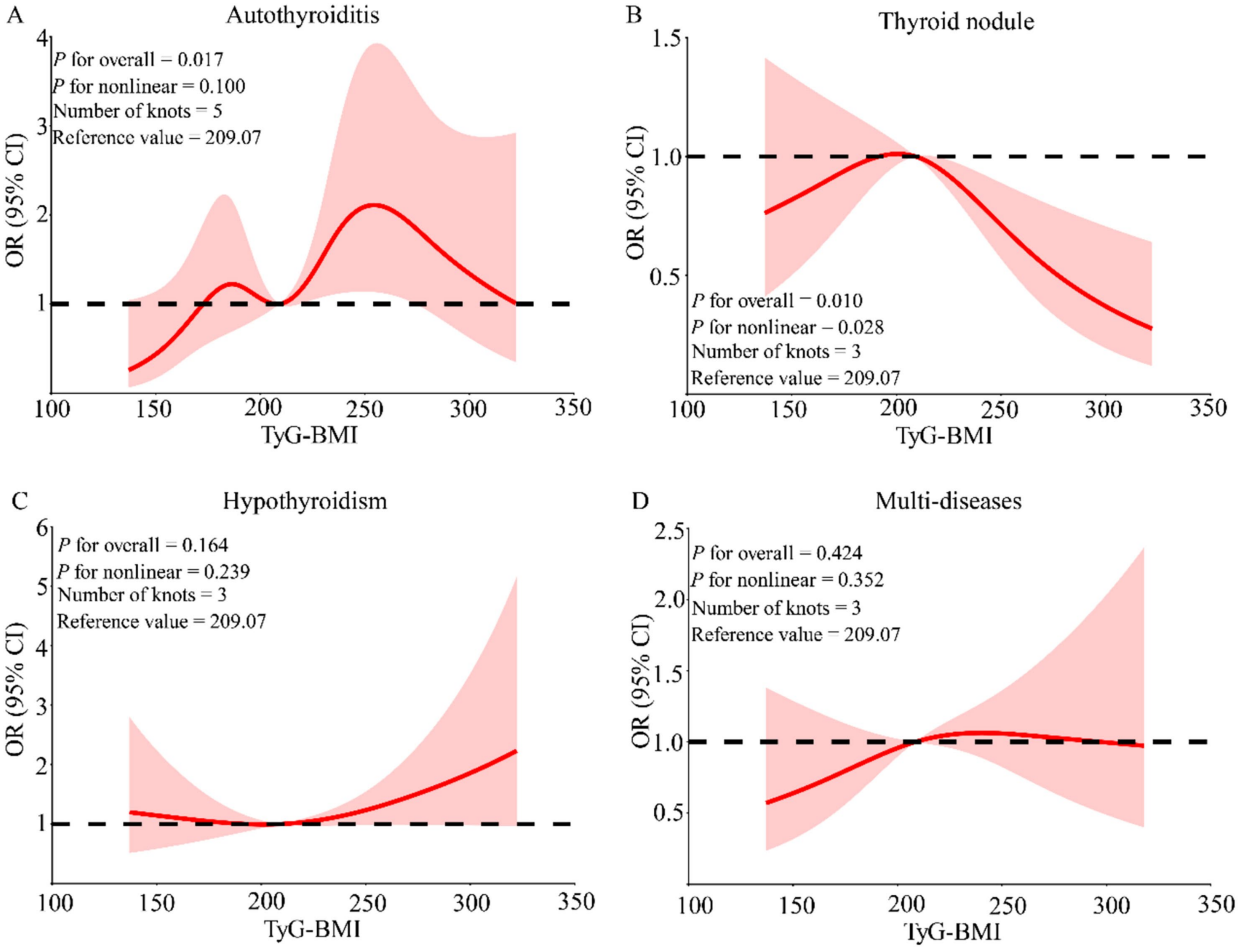
The nonlinear association between TyG-BMI index and thyroid diseases. The model adjusted for the following variables: age, gender, marital status, annual household income, education, and life scores. **(A)** Whether there is a nonlinear association between thyroiditis and TyG-BMI; **(B)** Whether there is a nonlinear association between thyroid nodule and TyG-BMI; **(C)** Whether there is a nonlinear association between hypothyroidism and TyG-BMI; **(D)** Whether there is a nonlinear association between multimorbidity and TyG-BMI; OR: odds ratio; 95% CI: 95% confidence interval; Multi-diseases: individuals with two or more thyroid diseases.

**Figure 4:**
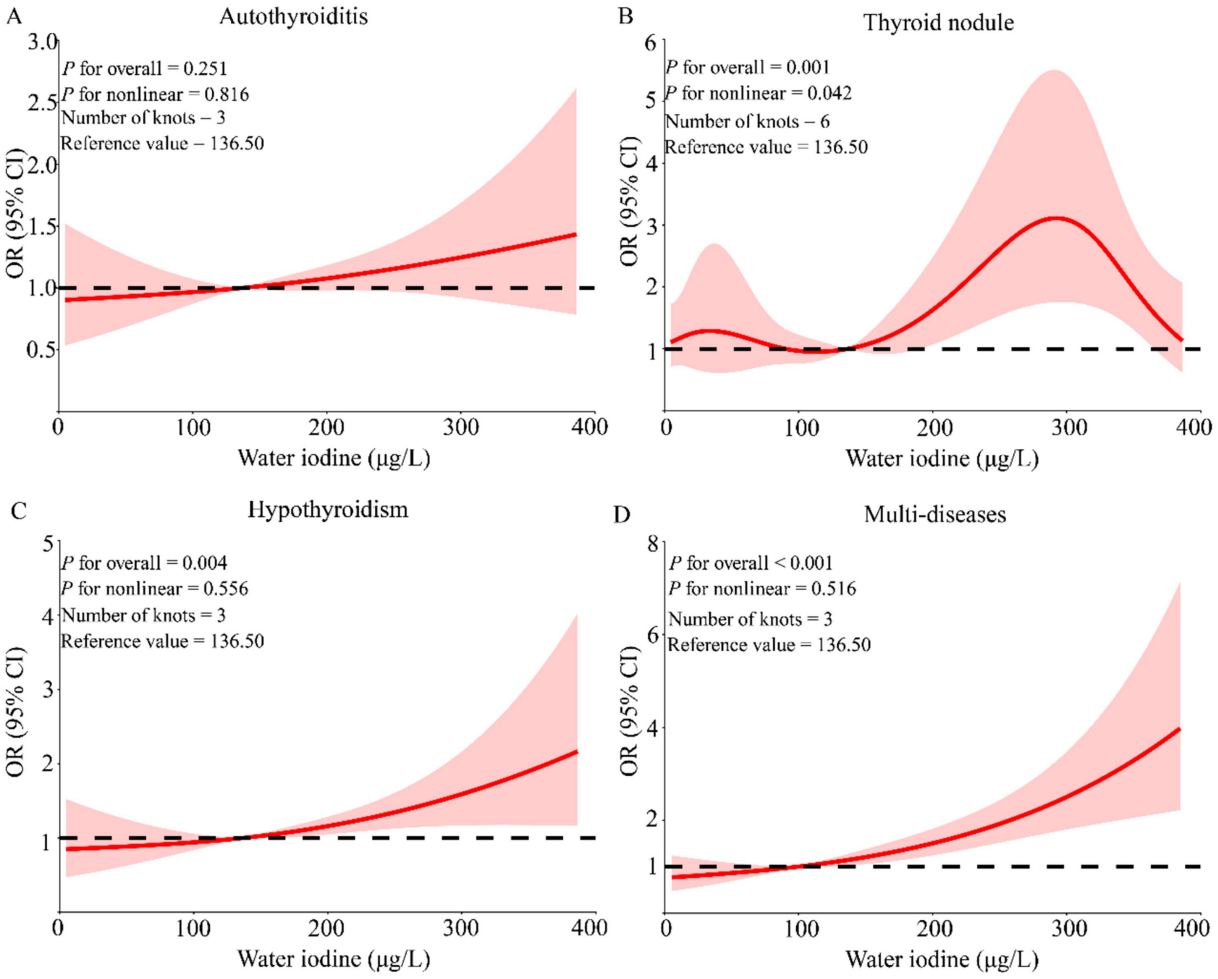
The nonlinear association between water iodine concentration and thyroid diseases. The model adjusted for the following variables: age, gender, marital status, annual household income, education, and life scores. **(A)** Whether there is a nonlinear association between thyroiditis and water iodine concentration; **(B)** whether there is a nonlinear association between thyroid nodule and water iodine concentration; **(C)** whether there is a nonlinear association between hypothyroidism and water iodine concentration; **(D)** whether there is a nonlinear association between multimorbidity and water iodine concentration. OR: Odds ratio; 95% CI: 95% confidence interval; Multi-diseases: Individuals with two or more thyroid diseases.

Previous linear association analyses between WI, TyG-BMI index, and thyroid diseases were conducted. To further investigate whether there was a nonlinear association, RCS regression was used to model this. After adjusting for age, gender, education, marital status, average annual income, and lifestyle score, a nonlinear association between thyroid nodules and TyG-BMI index was found (*P* for nonlinear = 0.028). No nonlinear associations were observed for the other diseases with TyG-BMI index (Figure 3). Similarly, after adjusting for covariates, A nonlinear association was also observed between WI and thyroid nodules (*P* for nonlinear = 0.042), with a U-shaped pattern indicating higher risks at both lower and higher WI levels (Figure 4).

### Predictive value of WI and TyG-BMI for thyroid diseases

This study added WI and TyG-BMI index to the baseline model and compared the fit of the two models using chi-square tests. The results showed that adding WI and TyG-BMI index significantly improved the fit of the models for three diseases and multiple concurrent thyroid diseases, with a reduction in Akaike information criterion (AIC) for the models, AIC is a standard for evaluating the goodness of fit of statistical models (Supplementary Table S8).

**Figure 5:**
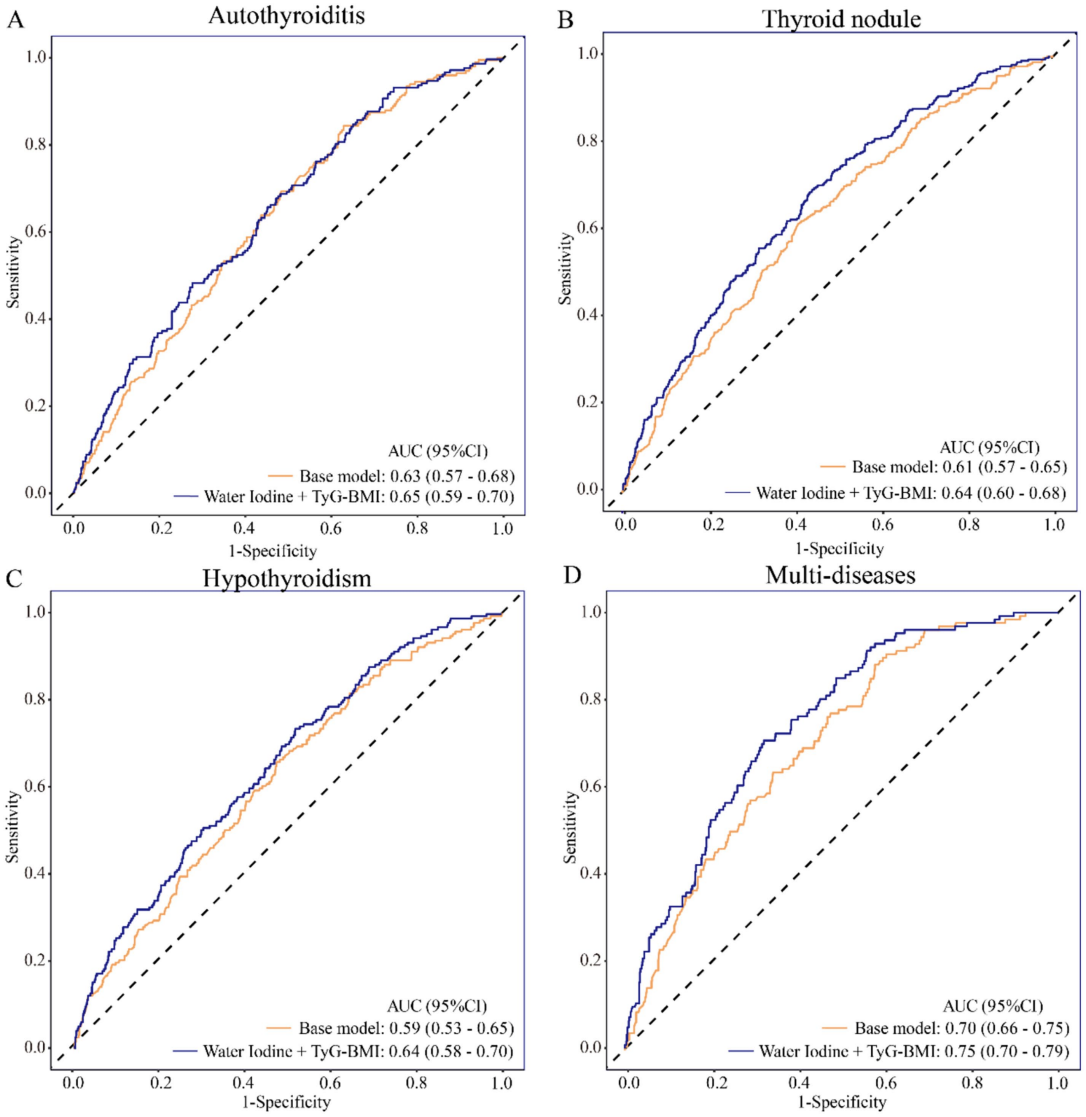
The predictive value of water iodine concentration and TyG-BMI index for thyroid diseases. **(A)** Predictive value of water iodine concentration and TyG-BMI for thyroiditis; **(B)** Predictive value of water iodine concentration and TyG-BMI for thyroid nodule; **(C)** Predictive value of water iodine concentration and TyG-BMI for hypothyroidism; **(D)** Predictive value of water iodine concentration and TyG-BMI for multiple thyroid diseases. The orange line represents the baseline model, which adjusted for age, gender, marital status, annual household income, education, and quality of life scores. The blue line represents the model that further adjusted for water iodine concentration and TyG-BMI on top of the baseline model. ROC curve: Receiver operating characteristic curve; AUC: area of under curve; OR: odds ratio; 95% CI: 95% confidence interval; Multi-diseases: individuals with two or more thyroid diseases.

To further assess the predictive value of combined WI and TyG-BMI, ROC curves and AUC changes were compared before and after adding WI and TyG-BMI index to the baseline model (The model adjusted for the following variables: age, gender, marital status, annual household income, education, and life scores.). Figure 5A shows that the model with added WI and TyG-BMI improved the predictive ability for thyroiditis, with AUC increasing from 0.63 to 0.65. Similarly, in the thyroid nodule model, AUC increased by 0.03 (from 0.61 to 0.64). In the hypothyroidism model, AUC increased by 0.05 (from 0.59 to 0.64). In the model for multiple concurrent thyroid diseases, AUC increased by 0.05 (from 0.70 to 0.75) (Figure 5). Additionally, DCA showed that the models with added WI and TyG-BMI were further away from the reference lines compared to the baseline models in the four models, indicating higher clinical value. Therefore, WI and TyG-BMI index further increased the predictive value of traditional risk factors, enhancing identification ability and risk reclassification (Supplementary Figure S2).

## Discussion

This cross-sectional study aimed to elucidate the linear and nonlinear associations between two key variables, WI, and TyG-BMI index, with the risk of thyroid diseases. The results highlighted the subtle ways in which these factors can interfere with and influence the assessment of thyroid disease risk, providing important insights for clinical practice and public health strategies. The study reconfirmed the association between WI in the environment and thyroid diseases. This study found that 982 individuals in the region had excess and deficiency iodine concentration in their drinking water, with 705 having excess levels of WI, indicating that most people are exposed to suboptimal iodine environments that may impact thyroid health. The mean, median and 95% reference range of TyG-BMI index in the total population were slightly higher than those in the population without thyroid diseases, suggesting a potential link between insulin resistance and thyroid dysfunction.

Descriptive statistics revealed that there were gender differences in disease prevalence. Females had higher prevalence of thyroiditis, thyroid nodules, and multiple thyroid diseases, which is consistent with previous studies and indicates that females are more susceptible to thyroid diseases (29, 30). Furthermore, differences in disease distribution based on educational level, lifestyle score, and water iodine concentration suggest a multifactorial nature. These findings indicate that sociodemographic and lifestyle factors play a significant role in the epidemiology of thyroid diseases (31). However, chi-square tests in the descriptive statistics did not show a statistically significant association between TyG-BMI and thyroid diseases, indicating that TyG-BMI did not exhibit a significant association with other thyroid diseases in univariate analysis. Nonetheless, multivariate models showed a significant association between TyG-BMI and thyroiditis, suggesting that TyG-BMI remains an important indicator for assessing the risk of thyroiditis even after considering potential confounding factors. Similarly, in Zhang et al.’s study, it was also found that the TyG index is a risk factor for the prevalence of positive thyroid antibodies, which further suggests a potential link between the TyG-BMI index and thyroid diseases (20).

The association analysis further demonstrated that excess WI and high TyG-BMI Q4 were positively associated with thyroiditis. In contrast, the data did not show significant associations between TyG-BMI and thyroid nodules, hypothyroidism, or multiple thyroid diseases. This result indicated that TyG-BMI had significant predictive value for thyroiditis but not for other thyroid diseases after adjusting for other factors. Although iodine deficiency could lead to hypothyroidism (32), this study found that excessive WI was a risk factor for hypothyroidism compared to adequate levels, suggesting that high iodine exposure increases the risk of hypothyroidism. This might be due to excessive iodine disrupting the synthesis process of thyroid hormones, inhibiting peroxidase activity, and reducing the thyroid’s oxidation of tyrosine, thereby decreasing the production of thyroid hormones such as thyroxin and triiodothyronine (33, 34). Another possible mechanism was the Wolff-Chaikoff effect, which was the temporary inhibition of thyroid function after short-term high iodine intake. This effect reduced internal uptake and organification of iodides by thyroid cells, further impacting thyroid hormone synthesis. Although the effect was usually temporary, in some cases, it can lead to persistent thyroid dysfunction (35–37).

Subgroup analyses provided specific insights into different genders and age groups. In males, excessive WI and Q4 levels of TyG-BMI were associated with higher odds of hypothyroidism, indicating that metabolic factors may be more significant in males. In contrast, excessive WI was highlighted as a risk factor for thyroid nodules and multiple thyroid diseases in females, suggesting greater sensitivity to environmental iodine exposure in females (38). Age-stratified analyses revealed that excessive WI was consistently associated with higher odds of thyroid nodules and hypothyroidism across age groups. Notably, among participants aged over 45 years, excessive WI was associated with even higher odds of multiple concurrent thyroid diseases. The age-related increase in the prevalence of thyroid nodules has been well documented in Chinese populations (39). Moreover, the elderly have been recognized as a susceptible population for iodine-induced thyroid dysfunction (40).

To further investigate the stability of this association, this study excluded participants with diabetes in the sensitivity analysis, as diabetes and thyroid dysfunction are closely interrelated. Existing research had shown that hypothyroidism and low-normal thyroid function are associated with an increased risk of diabetes, particularly in individuals with prediabetes (41). Furthermore, diabetes might lead to changes in thyroid function through alterations in gene expression and physiological abnormalities, thereby affecting glucose metabolism (28, 42).

To further explore the associations between WI and TyG-BMI and thyroid diseases, the study conducted interaction and mediation analyses. The present study found that water iodine and TyG-BMI exhibited an interaction effect on thyroiditis. Combined with existing evidence indicating that iodine nutrition can modulate insulin resistance-related metabolic pathways, this finding suggests that environmental and metabolic factors may synergistically influence thyroid susceptibility (43), and also underscores the complexity of thyroid disease pathogenesis, with environmental and metabolic factors interacting to influence disease development (4, 44). The present study identified a U-shaped association between water iodine concentration and the risk of thyroid nodules, with an inflection point at approximately 136.50 μg/L. This finding is consistent with the previously reported U-shaped relationship between urinary iodine and thyroid autoimmunity, and provides population-based quantitative evidence to inform monitoring thresholds in high-water-iodine areas (45). Currently, there is no dedicated research exploring the association between the TyG-BMI index and thyroid nodules. However, existing studies indicate that approximately 10–15% of thyroid nodules may progress to thyroid cancer (46, 47). Additionally, research shows that there is no significant correlation between BMI and the risk of thyroid cancer, with similar average BMI values observed in both cancer and benign nodule groups (48). Furthermore, studies have found that short-duration diabetes is significantly associated with a reduced risk of thyroid cancer, particularly in certain populations such as those over 40 years old, women, overweight or obese individuals, and those with a family history of diabetes. These findings suggest that the relationship between increased TyG-BMI and thyroid nodule risk is quite complex. Obesity may be linked to certain characteristics of thyroid cancer, while the short-term effects of diabetes may have a protective role in reducing the risk of thyroid cancer (49).

Finally, this study found that incorporating water iodine and the TyG-BMI index increased the AUC of the prediction model from 0.61 to 0.64, significantly reduced the AIC, and showed net benefit in decision curve analysis within a certain range of threshold probabilities. These findings indicate that water iodine and the TyG-BMI index provide additional, statistically significant predictive information for thyroid disease risk assessment beyond traditional risk factors. At the same time, this study acknowledges that the absolute improvement in AUC is modest, and the two decision curves showed near-complete overlap at lower threshold probabilities, indicating limited incremental net benefit in low-risk scenarios. This should be viewed in the context of the inherent complexity of thyroid disease pathogenesis, which involves a multifaceted interplay of genetic, environmental, metabolic, immunological, and nutritional factors. Given this complexity, perfect individual prediction based on a limited set of demographic, environmental, and metabolic variables is inherently challenging. Nevertheless, the predictive value of water iodine and the TyG-BMI index observed in this study is not negligible: they significantly improved model fit and provided meaningful net benefit at intermediate-to-high threshold probabilities, suggesting their potential utility in risk stratification of intermediate-to-high risk populations. Future efforts to refine predictive models may benefit from the inclusion of additional biomarkers, genetic susceptibility data, and more comprehensive dietary and environmental exposure assessments.

This study had certain strengths and limitations. Firstly, it conducted a comprehensive analysis of the association between WI and thyroid diseases, while also innovatively exploring the new metabolic indicator, TyG-BMI index, and its potential link with thyroid diseases. As a novel metabolic indicator, the TyG-BMI index may have had a certain association with thyroid diseases, and this study offered new insights into this area of exploration. Secondly, interaction analysis, mediation analysis, and RCS models were used to investigate the interactions and comprehensive associations between the two variables and thyroid disease. Finally, a predictive model was constructed to quantify the predictive effects of WI and TyG-BMI index on thyroid disease. However, this study has several limitations. First, it is a cross-sectional study, which limits its ability to establish causal associations. Second, there might have been recall bias in the collection of questionnaire data. Third, despite multi-stage random sampling, voluntary participation may introduce selection bias. Fourth, despite multivariable adjustment, residual confounding from unmeasured factors (such as dietary iodine intake) cannot be fully excluded.

## Conclusion

The study results indicate that WI and TyG-BMI index are associated with the presence of thyroid diseases, and that these new indicators are effective in predicting thyroid diseases. Therefore, in the prevention and control of thyroid diseases, a comprehensive approach combining environmental (WI) and individual factors (TyG-BMI index) should be taken. Public health measures focusing on controlling iodine intake and reducing metabolic abnormalities may help mitigate the risk of thyroid diseases and reduce the disease burden.

## Data Availability

The raw data supporting the conclusions of this article will be made available by the authors, without undue reservation.

## References

[1] Acosta GJ Singh Ospina N Brito JP. Epidemiologic changes in thyroid disease. Curr Opin Endocrinol Diabetes Obes. (2024) 31:184–90. doi: 10.1097/MED.0000000000000877 39087407

[2] Wang C Li Y Teng D Shi X Ba J Chen B . Hyperthyroidism prevalence in China after universal salt iodization. Front Endocrinol (Lausanne). (2021) 12:651534. doi: 10.3389/fendo.2021.651534, 34122333 PMC8194401

[3] Li Z Jia Z Zhou P He Q. Causal relationship between insomnia and thyroid disease: a bidirectional Mendelian randomization study. Brain Behav. (2024) 14:e70046. doi: 10.1002/brb3.70046, 39295101 PMC11410884

[4] Babić Leko M Gunjača I Pleić N Zemunik T. Environmental factors affecting thyroid-stimulating hormone and thyroid hormone levels. Int J Mol Sci. (2021) 22:6521. doi: 10.3390/ijms22126521, 34204586 PMC8234807

[5] Zimmermann MB Jooste PL Pandav CS. Iodine-deficiency disorders. Lancet. (2008) 372:1251–62. doi: 10.1016/S0140-6736(08)61005-3 18676011

[6] Winder M Kosztyła Z Boral A Kocełak P Chudek J. The impact of iodine concentration disorders on health and Cancer. Nutrients. (2022) 14:2209. doi: 10.3390/nu14112209, 35684009 PMC9182735

[7] Li J Li Y Shi X Teng D Teng X Teng W . Prevalence and risk factors of hypothyroidism after universal salt iodisation: a large cross-sectional study from 31 provinces of China. BMJ Open. (2023) 13:e064613. doi: 10.1136/bmjopen-2022-064613, 36854590 PMC9980360

[8] Yeo Y Han K Shin DW Kim D Jeong SM Chun S . Changes in smoking, alcohol consumption, and the risk of thyroid Cancer: a population-based Korean cohort study. Cancers (Basel). (2021) 13:2343. doi: 10.3390/cancers13102343, 34066228 PMC8150527

[9] Kim SJ Kim MJ Yoon SG Myong JP Yu HW Chai YJ . Impact of smoking on thyroid gland: dose-related effect of urinary cotinine levels on thyroid function and thyroid autoimmunity. Sci Rep. (2019) 9:4213. doi: 10.1038/s41598-019-40708-1, 30862792 PMC6414657

[10] Monaghan AM Mulhern MS McSorley EM Strain JJ Dyer M van Wijngaarden E . Associations between maternal urinary iodine assessment, dietary iodine intakes and neurodevelopmental outcomes in the child: a systematic review. Thyroid Res. (2021) 14:14. doi: 10.1186/s13044-021-00105-1, 34099006 PMC8182912

[11] Wang D Wan S Liu P Meng F Ren B Qu M . Associations between water iodine concentration and the prevalence of dyslipidemia in Chinese adults: a cross-sectional study. Ecotoxicol Environ Saf. (2021) 208:111682. doi: 10.1016/j.ecoenv.2020.111682, 33396014

[12] He J Lai Y Yang J Yao Y Li Y Teng W . The relationship between thyroid function and metabolic syndrome and its components: a cross-sectional study in a Chinese population. Front Endocrinol (Lausanne). (2021) 12:661160. doi: 10.3389/fendo.2021.661160, 33868183 PMC8044548

[13] Yao JY Liu P Zhang W Wang KW Lyu CP Zhang ZW . Obesity rather than metabolic syndrome is a risk factor for subclinical hypothyroidism and thyroid autoimmunity. Biomed Environ Sci. (2021) 34:819–23. doi: 10.3967/bes2021.111, 34782048

[14] Biondi B Kahaly GJ Robertson RP. Thyroid dysfunction and diabetes mellitus: two closely associated disorders. Endocr Rev. (2019) 40:789–824. doi: 10.1210/er.2018-00163, 30649221 PMC6507635

[15] Alwan H Ribero VA Efthimiou O Del Giovane C Rodondi N Duntas L. A systematic review and meta-analysis investigating the relationship between metabolic syndrome and the incidence of thyroid diseases. Endocrine. (2024) 84:320–7. doi: 10.1007/s12020-023-03503-7, 37688711 PMC11076217

[16] Kalra S Aggarwal S Khandelwal D. Thyroid dysfunction and type 2 diabetes mellitus: screening strategies and implications for management. Diabetes Ther. (2019) 10:2035–44. doi: 10.1007/s13300-019-00700-4, 31583645 PMC6848627

[17] Lillioja S Mott DM Spraul M Ferraro R Foley JE Ravussin E . Insulin resistance and insulin secretory dysfunction as precursors of non-insulin-dependent diabetes mellitus. Prospective studies of Pima Indians. N Engl J Med. (1993) 329:1988–92. doi: 10.1056/NEJM199312303292703, 8247074

[18] Yaribeygi H Farrokhi FR Butler AE Sahebkar A. Insulin resistance: review of the underlying molecular mechanisms. J Cell Physiol. (2019) 234:8152–61. doi: 10.1002/jcp.27603 30317615

[19] Zhou Z Liu Q Zheng M Zuo Z Zhang G Shi R . Comparative study on the predictive value of TG/HDL-C, TyG and TyG-BMI indices for 5-year mortality in critically ill patients with chronic heart failure: a retrospective study. Cardiovasc Diabetol. (2024) 23:213. doi: 10.1186/s12933-024-02308-w, 38902757 PMC11191322

[20] Zhang C Wang H Li Y Wang X Han Y Gao X . Association between the triglyceride-glucose index and thyroid disorders: a cross-sectional survey and Mendelian randomization analysis. Endocrine. (2024) 86:173–85. doi: 10.1007/s12020-024-03858-5, 38782862

[21] Guerrero-Romero F Simental-Mendía LE González-Ortiz M Martínez-Abundis E Ramos-Zavala MG Hernández-González SO . The product of triglycerides and glucose, a simple measure of insulin sensitivity. Comparison with the euglycemic-hyperinsulinemic clamp. J Clin Endocrinol Metab. (2010) 95:3347–51. doi: 10.1210/jc.2010-0288, 20484475

[22] Lim J Kim J Koo SH Kwon GC. Comparison of triglyceride glucose index, and related parameters to predict insulin resistance in Korean adults: an analysis of the 2007-2010 Korean National Health and nutrition examination survey. PLoS One. (2019) 14:e0212963. doi: 10.1371/journal.pone.0212963, 30845237 PMC6405083

[23] Er LK Wu S Chou HH Hsu LA Teng MS Sun YC . Triglyceride glucose-body mass index is a simple and clinically useful surrogate marker for insulin resistance in nondiabetic individuals. PLoS One. (2016) 11:e0149731. doi: 10.1371/journal.pone.0149731, 26930652 PMC4773118

[24] Wan S Qu M Wu H Ren B Jiang W Wang X . Autoimmune thyroid diseases after 25 years of universal salt iodisation: an epidemiological study of Chinese adults in areas with different water iodine levels. Br J Nutr. (2020) 124:853–64. doi: 10.1017/S0007114520001786, 32436480

[25] Huo RR Liao Q Zhai L You XM Zuo YL. Interacting and joint effects of triglyceride-glucose index (TyG) and body mass index on stroke risk and the mediating role of TyG in middle-aged and older Chinese adults: a nationwide prospective cohort study. Cardiovasc Diabetol. (2024) 23:30. doi: 10.1186/s12933-024-02122-4, 38218819 PMC10790273

[26] Xie H Pan H Qian T Hou X Zhao M Che W . Analysis of factors influencing prevalence and malignancy of thyroid nodules in various iodine uptake areas. Front Endocrinol (Lausanne). (2024) 15:1451911. doi: 10.3389/fendo.2024.1451911, 39574947 PMC11578705

[27] Feng X Wang F Yang W Zheng Y Liu C Huang L . Association between genetic risk, adherence to healthy lifestyle behavior, and thyroid Cancer risk. JAMA Netw Open. (2022) 5:e2246311. doi: 10.1001/jamanetworkopen.2022.46311, 36508215 PMC9856466

[28] Mohammed Hussein SM AbdElmageed RM. The relationship between type 2 diabetes mellitus and related thyroid diseases. Cureus. (2021) 13:e20697. doi: 10.7759/cureus.20697, 35106234 PMC8787293

[29] Søgaard M Farkas DK Ehrenstein V Jørgensen JO Dekkers OM Sørensen HT. Hypothyroidism and hyperthyroidism and breast cancer risk: a nationwide cohort study. Eur J Endocrinol. (2016) 174:409–14. doi: 10.1530/EJE-15-0989, 26863886

[30] Lin YP Iqbal U Nguyen PA Islam MM Atique S Jian WS . The concomitant Association of Thyroid Disorders and Myasthenia Gravis. Transl Neurosci. (2017) 8:27–30. doi: 10.1515/tnsci-2017-0006, 28729915 PMC5443889

[31] Wu Q Rayman MP Lv H Schomburg L Cui B Gao C . Low population selenium status is associated with increased prevalence of thyroid disease. J Clin Endocrinol Metab. (2015) 100:4037–47. doi: 10.1210/jc.2015-2222, 26305620

[32] Zimmermann MB Boelaert K. Iodine deficiency and thyroid disorders. Lancet Diabetes Endocrinol. (2015) 3:286–95. doi: 10.1016/S2213-8587(14)70225-6 25591468

[33] Farebrother J Zimmermann MB Andersson M. Excess iodine intake: sources, assessment, and effects on thyroid function. Ann N Y Acad Sci. (2019) 1446:44–65. doi: 10.1111/nyas.14041, 30891786

[34] Sohn SY Inoue K Rhee CM Leung AM. Risks of iodine excess. Endocr Rev. (2024) 45:858–79. doi: 10.1210/endrev/bnae019 38870258

[35] Eder S Hermann C Lamkowski A Kinoshita M Yamamoto T Abend M . A comparison of thyroidal protection by stable iodine or perchlorate in the case of acute or prolonged radioiodine exposure. Arch Toxicol. (2020) 94:3231–47. doi: 10.1007/s00204-020-02809-z, 32656655 PMC7415763

[36] Koukkou EG Roupas ND Markou KB. Effect of excess iodine intake on thyroid on human health. Minerva Med. (2017) 108:136–46. doi: 10.23736/S0026-4806.17.04923-0, 28079354

[37] Rump A Eder S Hermann C Lamkowski A Kinoshita M Yamamoto T . A comparison of thyroidal protection by iodine and perchlorate against radioiodine exposure in Caucasians and Japanese. Arch Toxicol. (2021) 95:2335–50. doi: 10.1007/s00204-021-03065-5, 34003340 PMC8241675

[38] Glinoer D. Pregnancy and iodine. Thyroid. (2001) 11:471–81. doi: 10.1089/105072501300176426 11396705

[39] Zhu Y Tong M Wang Y Liu Y Wang B Yang W . Prevalence of thyroid nodules and its association with water iodine among Chinese men and women. Environ Res. (2022) 212:113270. doi: 10.1016/j.envres.2022.113270, 35461842

[40] Leung AM Braverman LE. Consequences of excess iodine. Nat Rev Endocrinol. (2014) 10:136–42. doi: 10.1038/nrendo.2013.251, 24342882 PMC3976240

[41] Chaker L Ligthart S Korevaar TI Hofman A Franco OH Peeters RP . Thyroid function and risk of type 2 diabetes: a population-based prospective cohort study. BMC Med. (2016) 14:150. doi: 10.1186/s12916-016-0693-4, 27686165 PMC5043536

[42] Wang C. The relationship between type 2 diabetes mellitus and related thyroid diseases. J Diabetes Res. (2013) 2013:390534. doi: 10.1155/2013/390534 23671867 PMC3647563

[43] Zhao J Su Y Zhang JA Fang M Liu X Jia X . Inverse association between iodine status and prevalence of metabolic syndrome: a cross-sectional population-based study in a Chinese moderate iodine intake area. Diabetes Metab Syndr Obes. (2021) 14:3691–701. doi: 10.2147/DMSO.S322296, 34447259 PMC8384429

[44] Ferrari SM Fallahi P Antonelli A Benvenga S. Environmental issues in thyroid diseases. Front Endocrinol (Lausanne). (2017) 8:50. doi: 10.3389/fendo.2017.00050 28373861 PMC5357628

[45] Wang B He W Li Q Jia X Yao Q Song R . U-shaped relationship between iodine status and thyroid autoimmunity risk in adults. Eur J Endocrinol. (2019) 181:255–66. doi: 10.1530/EJE-19-0212 31252413

[46] Chung SR Baek JH Choi YJ Sung TY Song DE Kim TY . The relationship of thyroid nodule size on malignancy risk according to histological type of thyroid cancer. Acta Radiol. (2020) 61:620–8. doi: 10.1177/0284185119875642, 31554409

[47] Yun KJ Ha J Kim MH Seo YY Kim MK Kwon HS . Comparison of natural course between thyroid Cancer nodules and thyroid benign nodules. Endocrinol Metab (Seoul). (2019) 34:195–202. doi: 10.3803/EnM.2019.34.2.195, 31257747 PMC6599907

[48] Paes JE Hua K Nagy R Kloos RT Jarjoura D Ringel MD. The relationship between body mass index and thyroid cancer pathology features and outcomes: a clinicopathological cohort study. J Clin Endocrinol Metab. (2010) 95:4244–50. doi: 10.1210/jc.2010-0440, 20519347 PMC2936072

[49] Wang M Gong WW Lu F Hu RY He QF Yu M. The association between diabetes and thyroid cancer risk: a hospital-based case-control study in China. BMC Endocr Disord. (2021) 21:21. doi: 10.1186/s12902-021-00684-y, 33509182 PMC7845043

