# Supplementary material for: Analysis of the association between water iodine concentration and TyG-BMI index with thyroid diseases: a cross-sectional study in Shandong province, China
Source: Front Nutr. 2026 May 13;13:1819662. doi: 10.3389/fnut.2026.1819662 (PMC13212242; doi:10.3389/fnut.2026.1819662)
Supplement: Supplementary file 1 [file Table_1.DOCX]

**Analysis of the association between water iodine concentration and TyG-BMI index with thyroid diseases: a cross-sectional study in Shandong province, China**

Chun-Hu Li^1^, Meng Zhao^1^,Tong Zhao^1^, Yu-Hang Liu^1^, Zong-Yu Yue^1^, Zhe-Xu Zhang^1^, Xiang-Kun Zeng^1^, Dian-Jun Sun^1^, Peng Liu^1^*

1. Harbin Medical University, Center for Endemic Disease Control, Harbin, Heilongjiang Province, China, 150081.

Email: liup7878@163.com

**STable1** Variable assignment table.

| Variables | Assignment |
| --- | --- |
| Sex | Male = “0”; Female = “1” |
| Annual household income | <5000 = “0”; 5000-10000 = “1”; 10000-30000 = “2”;  30000-50000 = “3”; >50000 = “4” |
| Educational attainment | No = “0”; Elementary school = “1”; Middle School = “2”;  High School = “3”; University = “4” |
| Marital status | Unmarried = “0”; Married = “1”; Divorced = “2”; |
| Lifestyle scores | Poor = “0”; Medium = “1”; Excellent = “2” |
| Water iodine | Deficiency: <40 μg/L = “1”; Adequate: 40-100 μg/L = “0”; Excess: >100 μg/L = “2” |
| Hypothyroidism | Yes = “1”; No = “0” |
| Hyperthyroidism | Yes = “1”; No = “0” |
| Thyroiditis | Yes = “1”; No = “0” |
| Thyroid nodules | Yes = “1”; No = “0” |
| Thyroid diseases | Yes = “1”; No = “0” |

**STable 2** Lifestyle score composition

| Lifestyle score composition | Source and definition | Score |
| --- | --- | --- |
| 1. Smoke | Never and previous | 1 |
|  | Current | 0 |
| 2. Moderate alcohol consumption | Women: <14g/day 1 drink-equivalent | 1 |
|  | Men: <28g/day 2 drink-equivalent | 1 |
|  | Women>=14 1 drink-equivalent | 0 |
|  | Men>=28 2 drink-equivalent | 0 |
| 3. Diet index | Pure meat diet | 0 |
|  | Vegetarian diet | 0 |
|  | Mixed diet | 1 |
| 4. Total moderate-vigorous physical activity | >=150 minutes moderate activity per week or >= 75 minutes vigorous activity per week | 1 |
|  | Other | 0 |
| 5. Be a Weight | BMI (kg/m^2^) |  |
|  | 18.5-24.9 | 1 |
|  | 25-29.9 | 0 |
|  | >=30 or <18.5 | 0 |
|  | Waist circumference (cm(in)) |  |
|  | Men: <94 (<37) | 1 |
|  | Women: <80 (<31.5) | 1 |
|  | Men: 94-<102 (37-<40) | 0 |

**STable 3** Number of people with different types of thyroid disease.

| Thyroid disease | Number | | |
| --- | --- | --- | --- |
|  | Male | Female | ALL |
| Single-disease |  |  |  |
| Thyroiditis | 16 | 103 | 119 |
| Thyroid nodules | 40 | 205 | 245 |
| Hypothyroidism | 19 | 84 | 103 |
| Hyperthyroidism | 2 | 4 | 6 |
| Multi diseases |  |  |  |
| Thyroiditis+ Hypothyroidism | 3 | 47 | 50 |
| Thyroiditis+ Hyperthyroidism | 1 | 7 | 8 |
| Thyroiditis+ Thyroid nodules | 3 | 22 | 25 |
| Thyroid nodules+ Hypothyroidism | 1 | 45 | 46 |
| Thyroid nodules+ Hyperthyroidism | 0 | 3 | 3 |
| Thyroiditis+ Thyroid nodules + Hypothyroidism | 0 | 2 | 2 |
| Thyroiditis+ Thyroid nodules + Hyperthyroidism | 0 | 1 | 1 |

Single-disease: Individuals with a single thyroid disease; Multi-diseases: Individuals with two or more thyroid diseases.

**STable 4** Distribution of TyG-BMI index in different populations.

|  | Q_2.5_ | Q_25_ | Q_50_ | Q_75_ | Q_97.5_ | Mean |
| --- | --- | --- | --- | --- | --- | --- |
| TyG-BMI in all population | 146.37 | 184.24 | 209.35 | 239.19 | 306.72 | 213.488 |
| Kurtosis test | Kurtosis test=3.736, *P*<0.001 | | | | | |
| Skewness test | Skewness test=0.588, *P*<0.001 | | | | | |
| Normality test | Kolmogorov-Smirno normality test=0.047, *P*<0.001 | | | | | |
| TyG-BMI in normal population | 143.17 | 182.10 | 207.68 | 235.80 | 304.23 | 211.93 |
| Kurtosis test | Kurtosis test=4.049, *P*<0.001 | | | | | |
| Skewness test | Skewness test=0.700, *P*<0.001 | | | | | |
| Normality test | Kolmogorov-Smirno normality test=0.051, *P*<0.001 | | | | | |

The distribution of TyG-BMI in the total population and the normal population. Q_2.5_ represents the 2.5th percentile; Q_25_ represents the 25th percentile; Q_50_ represents the 50th percentile (median); Q_75_ represents the 75th percentile; Q_97.5_ represents the 97.5th percentile;

**STable 5** Association between water iodine concentration and TyG-BMI index with Positive TPOAb/TGAb.

| Variables |  | Positive TPOAb/TGAb |
| --- | --- | --- |
|  |  | OR (95% CI) |
| Water Iodine | Adequate |  |
|  | Deficiency | 0.82 (0.50-1.36) |
|  | Excess | 1.37 (0.93-2.03) |
| TyG- BMI | Q1 |  |
|  | Q2 | 1.27 (0.78-2.04) |
|  | Q3 | 1.53 (0.95-2.48) |
|  | Q4 | **1.96 (1.23-3.14)** |

The model was adjusted for age, gender, marital status, annual household income, education, and quality of life scores. OR: Odds Ratio; 95%CI: 95% Confidence Interval; Q1-Q4 represent the four quartiles of the TyG-BMI distribution.

**STable 6** Stratified analysis of the association between water iodine concentration, TyG-BMI index and thyroid diseases.

| Variables |  |  | OR (95% CI) | | | |
| --- | --- | --- | --- | --- | --- | --- |
|  |  |  | Autothyroiditis | Thyroid nodule | Hypothyroidism | Multi-diseases |
| Sex |  |  |  |  |  |  |
| Male | Water Iodine | Adequate |  |  |  | - |
|  |  | Deficiency | 4.36 (0.84-22.53) | 0.55 (0.21-1.48) | 1.96 (0.33-11.60) | - |
|  |  | Excess | 3.92 (0.80-19.07) | 1.00 (0.46-2.19) | **5.89 (1.26-27.58)** | - |
|  | TyG-BMI | Q1 |  |  |  | - |
|  |  | Q2 | 2.29 (0.52-10.00) | 0.93 (0.35-2.47) | 1.33 (0.27-6.56) | - |
|  |  | Q3 | 1.57 (0.35-7.16) | 1.40 (0.55-3.56) | 1.88 (0.39-9.08) | - |
|  |  | Q4 | 2.54 (0.58-11.12) | 1.06 (0.39-2.87) | **5.64 (1.37-23.24)** | **-** |
| Female | Water Iodine | Adequate |  |  |  | - |
|  |  | Deficiency | 1.00 (0.51-1.98) | 1.50 (0.88-2.55) | 0.92 (0.43-1.99) | 1.00 (0.48-2.08) |
|  |  | Excess | 1.36 (0.76-2.45) | **2.10 (1.32-3.35)** | 1.45 (0.76-2.75) | **2.81 (1.54-5.15)** |
|  | TyG- BMI | Q1 |  |  |  |  |
|  |  | Q2 | 1.28 (0.65-2.52) | 1.18 (0.75-1.87) | 0.60 (0.29-1.25) | 1.45 (0.76-2.78) |
|  |  | Q3 | 1.86 (0.96-3.60) | 0.84 (0.52-1.37) | 0.85 (0.42-1.71) | 1.35 (0.70-2.62) |
|  |  | Q4 | **2.30 (1.19-4.44)** | 0.72 (0.43-1.19) | 1.14 (0.58-2.24) | 1.39 (0.71-2.72) |
| Age |  |  |  |  |  |  |
| ≤45 | Water Iodine | Adequate |  |  |  |  |
|  |  | Deficiency | 1.45 (0.61-3.45) | 2.10 (0.81-5.43) | 2.12 (0.57-7.83) | 0.34 (0.11-1.11) |
|  |  | Excess | 1.13 (0.48-2.65) | **4.03 (1.66-9.82)** | **4.05 (1.19-13.74)** | 1.96 (0.79-4.85) |
|  | TyG-BMI | Q1 |  |  |  |  |
|  |  | Q2 | 1.01 (0.40-2.53) | 1.15 (0.57-2.31) | 0.56 (0.21-1.48) | 0.94 (0.32-2.76) |
|  |  | Q3 | 1.13 (0.54-2.37) | 1.37 (0.69-2.70) | 0.65 (0.25-1.65) | 1.49 (0.53-4.22) |
|  |  | Q4 | 1.24 (0.51-2.99) | 0.81 (0.38-1.70) | 1.19 (0.52-2.70) | 2.01 (0.69-5.86) |
| >45 | Water Iodine | Adequate |  |  |  |  |
|  |  | Deficiency | 1.17 (0.47-2.91) | 2.04 (0.78-5.37) | 2.11 (0.56-8.04) | 1.72 (0.70-4.25) |
|  |  | Excess | 0.87 (0.35-2.12) | **4.19 (1.69-10.38)** | **3.83 (1.10-13.34)** | **3.13 (1.45-6.75)** |
|  | TyG- BMI | Q1 |  |  |  |  |
|  |  | Q2 | 1.11 (0.42-2.88) | 1.15 (0.55-2.42) | 0.37 (0.12-1.13) | 0.89 (0.41-1.96) |
|  |  | Q3 | 1.11 (0.43-2.85) | 1.54 (0.74-3.23) | 0.56 (0.20-1.53) | 1.36 (0.64-2.89) |
|  |  | Q4 | 1.85 (0.75-4.54) | 0.88 (0.40-1.94) | 1.17 (0.48-2.83) | 1.32 (0.63-2.77) |

The model was adjusted for age, gender, marital status, annual household income, education, and quality of life scores. OR: Odds Ratio; 95%CI: 95% Confidence Interval; Mul-disease: Individuals with two or more thyroid diseases; Q1-Q4 represent the four quartiles of the TyG-BMI distribution; Multi-diseases: Individuals with two or more thyroid diseases.

**STable 7** Sensitivity analysis with separate adjustment for individual lifestyle factors.

| Variables |  | OR (95% CI) | | | |
| --- | --- | --- | --- | --- | --- |
|  |  | Autothyroiditis | Thyroid nodule | Hypothyroidism | Multi-diseases |
| Water Iodine | Adequate |  |  |  | - |
|  | Deficiency | 1.38 (0.72 ~ 2.63) | 1.21 (0.76 ~ 1.93) | 1.11 (0.54 ~ 2.25) | 1.32 (0.90 ~ 1.92) |
|  | Excess | **1.80 (1.01 ~ 3.19)** | **1.76 (1.18 ~ 2.63)** | **1.88 (1.02 ~ 3.44)** | **2.37 (1.70 ~ 3.30)** |
| TyG-BMI | Q1 |  |  |  |  |
|  | Q2 | 1.66 (0.87 ~ 3.17) | 1.33 (0.86 ~ 2.06) | 0.57 (0.29 ~ 1.13) | 1.30 (0.89 ~ 1.90) |
|  | Q3 | **2.35 (1.16 ~ 4.75)** | 1.19 (0.72 ~ 1.97) | 0.73 (0.36 ~ 1.50) | 1.48 (0.96 ~ 2.26) |
|  | Q4 | **4.02 (1.70 ~ 9.52)** | 1.05 (0.55 ~ 2.03) | 1.10 (0.47 ~ 2.60) | **1.97 (1.14 ~ 3.39)** |

The model was adjusted for age, gender, marital status, annual household income, education, smoking, drinking, BMI, diet index, physical activity.

**STable 8** Incremental analysis of water iodine concentration and TyG-BMI for a model of thyroid diseases.

| Variables | Autothyroiditis | | Thyroid nodule | | Hypothyroidism | | Multi-diseases | |
| --- | --- | --- | --- | --- | --- | --- | --- | --- |
|  | AIC | χ^2^ | AIC | χ^2^ | AIC | χ^2^ | AIC | χ^2^ |
| Base model ^a^ | 778.19 |  | 1201.97 |  | 717.30 |  | 675.27 |  |
| Water Iodine + TyG-BMI | **774.94** | **12.25*** | 1198.34 | **13.62*** | 712.15 | **15.15**** | 660.30 | **24.97***** |

a: The model was adjusted for age, gender, marital status, annual household income, education, and quality of life scores. AIC: Akaike Information Criterion; *: *P*<0.05; **: *P*<0.01; ***: *P*<0.001; Multi-diseases: Individuals with two or more thyroid diseases.


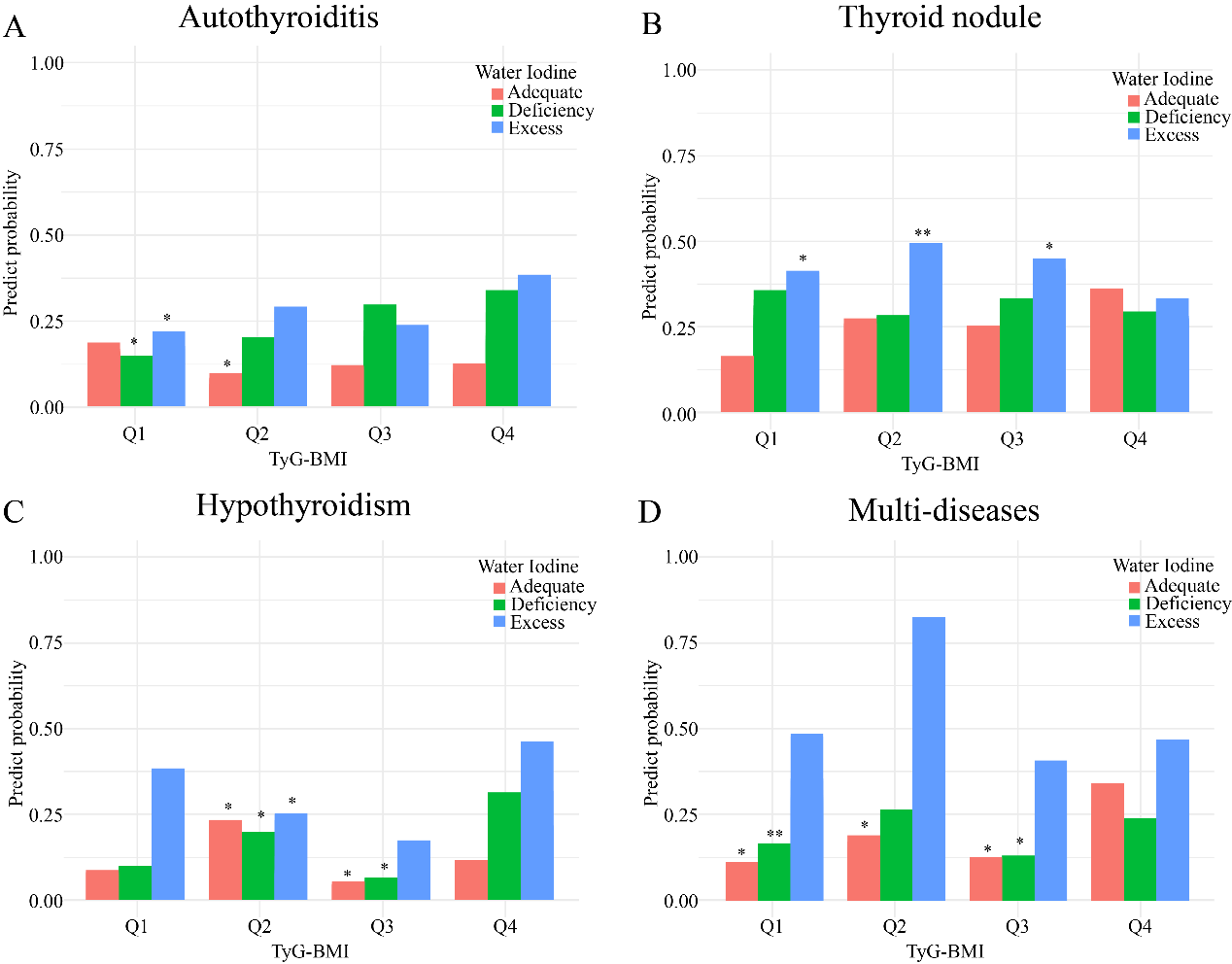


**SFigure 1** Visualization of the interaction between water iodine concentration and TyG-BMI index in thyroid diseases.

The model was adjusted for age, gender, marital status, annual household income, education, and quality of life scores. Q1-Q4 represent the four quartiles of the TyG-BMI distribution; Multi-diseases: Individuals with two or more thyroid diseases. *: *P*<0.05, **: *P*<0.01, ***: *P*<0.001.


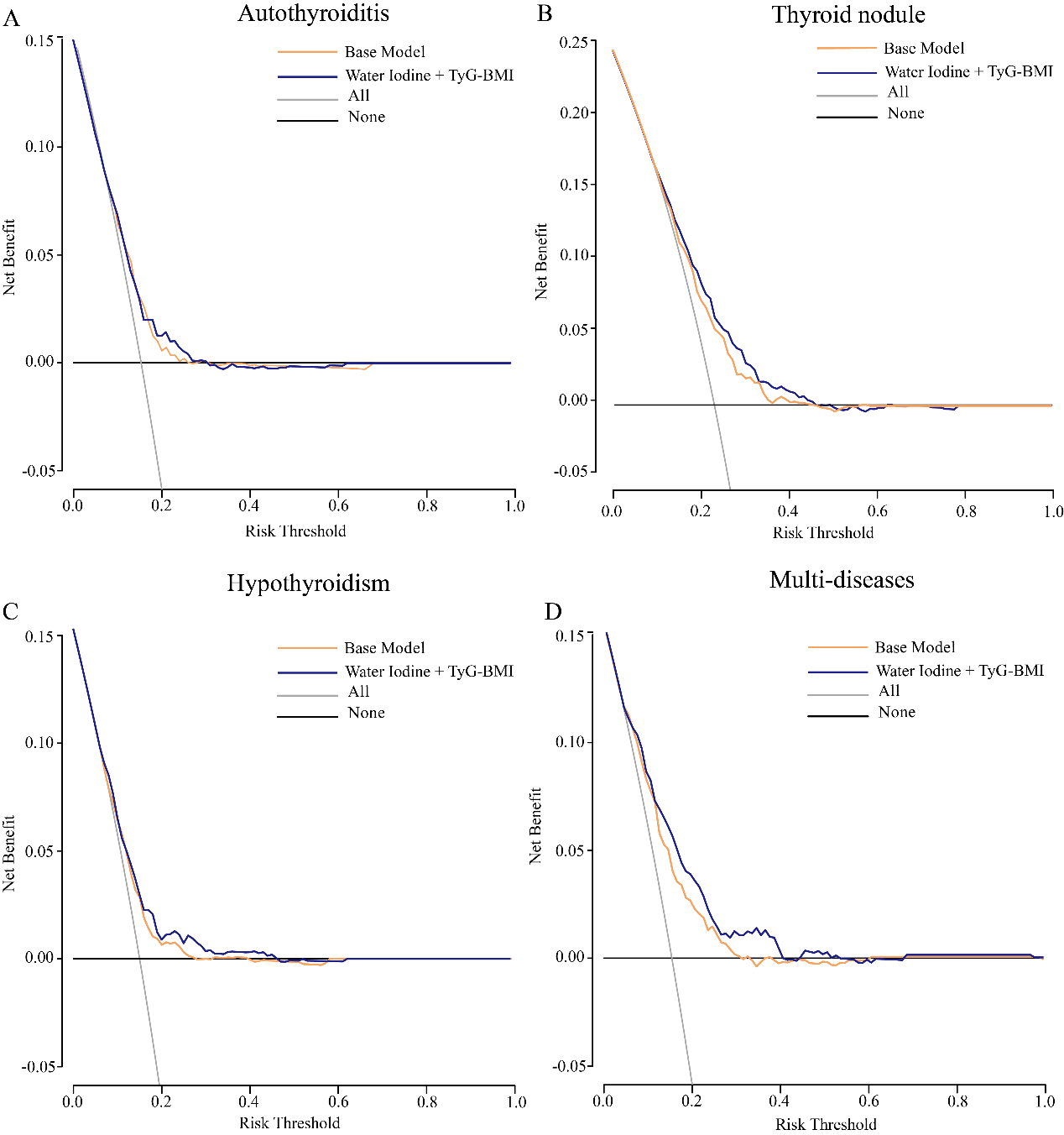


**SFigure 2** Decision analysis curves of water iodine concentration and TyG-BMI for thyroid diseases.

A: Decision curve for thyroiditis; B: Decision curve for thyroid nodules; C: Decision curve for hypothyroidism; D: Decision curve for multiple diseases. The orange line represents the baseline model, which adjusted for age, gender, marital status, annual household income, education, and quality of life scores.

The blue line represents the model that further adjusted for water iodine concentration and TyG-BMI on top of the baseline model. Multi-diseases: Individuals with two or more thyroid diseases.
